# Low-frequency autoregulation index for calculation of optimal cerebral perfusion pressure in severe traumatic brain injury

**DOI:** 10.1186/cc12265

**Published:** 2013-03-19

**Authors:** F Guiza, G Meyfroidt, M Schuhmann, G Van den Berghe, I Piper, B Depreitere

**Affiliations:** 1 University Hospitals Leuven, Belgium; 2 University Hospital Tübingen, Germany; 3 Southern General Hospital, Glasgow, UK

## Introduction

Traumatic brain injury (TBI) is a major cause of permanent disability and death in young patients. Controversy exists regarding the optimal cerebral perfusion pressure (CPP) required in TBI management. A tool for monitoring autoregulation and determining an optimal CPP is the pressure reactivity index (PRx), defined as a moving correlation coefficient between the mean arterial blood pressure (MAP) and intracranial pressure (ICP) at a frequency of at least 60 Hz. This requirement of high frequency has constrained its use to a few academic centers. An association was shown between outcome and continuous optimal CPP based on 4 hours of PRx [1]. We present a novel low-frequency autoregulation index (LAx), based on correlations between ICP and MAP at a standard minute-by-minute time resolution.

## Methods

A total of 182 patients from the Brain-IT [2] multicentre European database had registered outcome and ICP and MAP for the first 48 ICU hours. Twenty-one TBI patients admitted to the university hospitals of Leuven, Belgium and Tubingen, Germany were continuously monitored using ICM+ software (Cambridge Enterprise) allowing for continuous PRx calculation. Autoregulation indices versus CPP plots for PRx and LAx were computed to determine optimal CPP every minute during the first 48 ICU hours [1].

## Results

On the Brain-IT database, LAx resulted in an optimal CPP for 90% of the first 48 hours. Table 1 shows recommendations with respect to outcome. In the Leuven-Tübingen database, PRx and LAx resulted in 44% and 92% recommendations respectively. The average difference between methods was 5.26 mmHg.

## Conclusion

The differences in optimal CPPs derived from PRx and LAx were not clinically significant. LAx allowed for recommendations to be computed for longer periods. Significantly better outcome (Table 1) was observed in patients for whom optimal CPP derived from LAx was maintained.

**Table 1:** 

	Nonsurvivors	Survivors	*P *value
Time within CPPopt-%	18.3 (15.5; 24.4)	24.8 (19.7; 28.7)	0.0004
|CPP-CPPopt| (mmHg)	7.05 (4.98; 9.73)	5.37 (4.49; 6.82)	0.0024
